# A cost-effectiveness evaluation of a dietitian-delivered telephone coaching program during pregnancy for preventing gestational diabetes mellitus

**DOI:** 10.1186/s12962-024-00520-9

**Published:** 2024-03-01

**Authors:** Susan de Jersey, Syed Afroz Keramat, Angela Chang, Nina Meloncelli, Taylor Guthrie, Elizabeth Eakin, Tracy Comans

**Affiliations:** 1 Department of Dietetics and Food Services, Royal Brisbane and Women’s Hospital, Metro North Health, Brisbane, Australia; 2 Centre for Health Services Research, Faculty of Medicine, The University of Queensland, Brisbane, Australia; 3 Centre for Allied Health Research, Royal Brisbane and Women’s Hospital, Metro North Health, Brisbane, Australia; 4 School of Public Health, Faculty of Medicine, The University of Queensland, Brisbane, QLD Australia

**Keywords:** GDM, Prevention, Cost-effectiveness, Nutrition

## Abstract

**Background:**

This study aimed to evaluate the cost-effectiveness of a telehealth coaching intervention to prevent gestational diabetes mellitus (GDM) and to calculate the breakeven point of preventing GDM.

**Methods:**

Data to inform the economic evaluation model was sourced directly from the large quaternary hospital in Brisbane, where the Living Well during Pregnancy (LWdP) program was implemented, and further supplemented with literature-based estimates where data had not been directly collected in the trial. A cost-effectiveness model was developed using a decision tree framework to estimate the potential for cost savings and quality of life improvement. A total of 1,315 pregnant women (49% with a BMI 25-29.9, and 51% with a BMI ≥ 30) were included in the analyses.

**Results:**

The costs of providing routine care and routine care plus LWdP coaching intervention to pregnant women were calculated to be AUD 20,933 and AUD 20,828, respectively. The effectiveness of the LWdP coaching program (0.894 utility) was slightly higher compared to routine care (0.893). Therefore, the value of the incremental cost-effectiveness ratio (ICER) was negative, and it indicates that the LWdP coaching program is a dominant strategy to prevent GDM in pregnant women. We also performed a probabilistic sensitivity analysis using Monte Carlo simulation through 1,000 simulations. The ICE scatter plot showed that the LWdP coaching intervention was dominant over routine care in 93.60% of the trials using a willingness to pay threshold of AUD 50,000.

**Conclusion:**

Findings support consideration by healthcare policy and decision makers of telehealth and broad-reach delivery of structured lifestyle interventions during pregnancy to lower short-term costs associated with GDM to the health system.

**Supplementary Information:**

The online version contains supplementary material available at 10.1186/s12962-024-00520-9.

## Background

Excessive gestational weight gain (GWG) occurs in 40 to 60% of pregnancies [1], especially for the 50% of women who commence their pregnancy classified above a healthy weight [2]. Weight gain above recommendations increases the risk of many adverse health outcomes, particularly gestational diabetes mellitus (GDM), and large-for-gestational-age babies [1]. Increased adverse outcomes during pregnancy contribute significantly to the cost of antenatal health care provision [3]. Long-term, women and their infants are also more likely to develop complications arising from the development of obesity and diabetes [4], with associated healthcare costs [5].

GDM affects around 15% of pregnancies in Australia and is a common and potentially serious pregnancy complication which is more common in those with excess GWG [6]. GDM is persistent, elevated blood glucose levels first detected in pregnancy which can result in birthing and neonatal complications [7]. A meta-analysis has shown that excessive GWG increases the risk of GDM by a factor of 1.4, regardless of pre-pregnancy body mass index [8]. The economic burden of treating GDM greatly increases the costs to the health system of providing pregnancy care [9, 10], with women also bearing financial costs of treatment through blood glucose monitoring equipment, increased healthcare appointments and medication [11].

Recommendations for healthy GWG developed by the Institute of Medicine (IOM) [4] are included in several pregnancy care guidelines internationally [12] including Australia [13]. Interventions that include dietary counselling, physical activity and weight monitoring have successfully reduced GWG in research and clinical settings [14–17], and benefit individual outcomes [16, 18], including prevention of GDM [17–19]. Additionally, lifestyle interventions in pregnancy appear to be cost-effective with good return on investment [20].

However, women who start pregnancy above a healthy weight may experience greater barriers to achieving healthy weight gain and require more intensive support [21]. Furthermore, traditional models of face-to-face antenatal support (beyond routine care), including dietetic appointments, have limited uptake or are poorly attended [22–25].

The Living Well during Pregnancy (LWdP) program is a dietitian-delivered telephone coaching service for women at risk of excessive GWG [26], which was associated with improvements in dietary and physical activity behaviours [27]. This study performed an economic evaluation of the program as a potential intervention to prevent GDM.

## Methods

### Aims

The aim of this study was to calculate the cost-effectiveness of the LWdP coaching intervention to prevent GDM. A secondary aim was to calculate the breakeven point of preventing GDM.

### Study design and setting

The LWdP was implemented into usual care at a quaternary metropolitan hospital in 2018. A detailed description of program and its planned evaluation (including a cost-effectiveness evaluation) are available in the published protocol [26]. The evaluation outcomes completed as a hybrid effectiveness-implementation study has been reported elsewhere [28].

### The Living Well during Pregnancy program (LWdP)

The LWdP program was delivered in addition to routine antenatal care for those women who were referred or self-refer. The program aimed to support pregnant women at high-risk of excess GWG to track within their recommended weight gain range for their pre-pregnancy body mass index (BMI) based on IOM recommendations [4] through changing eating and activity behaviours [26]. This included healthy eating, and physical activity, consistent with dietary and physical activity guidelines for pregnancy [26]. Women were eligible for up to 10 telephone coaching calls over their pregnancy and were provided with a participant workbook. Accredited Practising Dietitians with experience in providing antenatal care services who had undergone additional training in motivational interviewing were trained specifically for the delivery of the program [26]. A key component of the program was continuity of care through allocation of the same Dietitian throughout the duration of the program. The program was adapted for pregnancy from the Healthy Living after Cancer program [29].

### Routine antenatal care

In Australia, pregnant women birthing in the public system receive free antenatal care provided by midwives, shared care with a general practitioner, obstetrician or a combination of these [30].

### Data

Data to inform the economic evaluation model was sourced directly from the large quaternary hospital in Brisbane, where LWdP was implemented and further supplemented with literature-based estimates where data had not been directly collected in the trial. The hospital manages 4500 births per year. Details of maternal anthropometry (height, pre-pregnancy weight, BMI, weight at 36 weeks), pregnancy complications such as GDM, onset of labour, mode of delivery and foetal outcomes (gender, weight, outcomes, head circumference, APGAR scores, neonatal nursery admissions, special care nursery admissions and congenital abnormalities) were sought from the clinical data systems. Information regarding admission date/time, discharge date/time, AR-DRG, International Classification of Diseases 10 (ICD-10) including diagnosis and procedure codes, Australian Classification of Health Interventions (ACHI) codes and cost were sought from the Health Funding and Analysis Unit of the participant Hospital and Health Service costing systems.

### Economic evaluation

A cost-effectiveness analysis using hospital data from 2020 was performed using a decision tree framework to estimate the potential for cost savings and quality of life improvement measured in Quality Adjusted Life Years (QALYs) from the wider implementation of the LWdP coaching program. A decision tree model was built in TreeagePro® to compare the coaching intervention to an alternative strategy of routine care to women with a BMI of 25 kg/m^2^ or greater, or who gain weight too quickly during early pregnancy in order to prevent GDM.

Women were classified as underweight (BMI < 18.5 kg/m^2^), normal weight (18.5-24.9 kg/m^2^), overweight (25-29.9 kg/m^2^) or obese (≥ 30 kg/m^2^) according to the World Health Organization [31]. Women classified as healthy weight and underweight were not included in the model as these women were not the primary focus of the intervention. Termination of pregnancy (spontaneous or planned) was also not included in the model as the majority occur prior to the intervention commencing. Stillbirth was excluded as these were < 1% of all births and did not have a material effect on the results of the model (tested in structural uncertainty prior to final model specification).

**Fig. 1:**
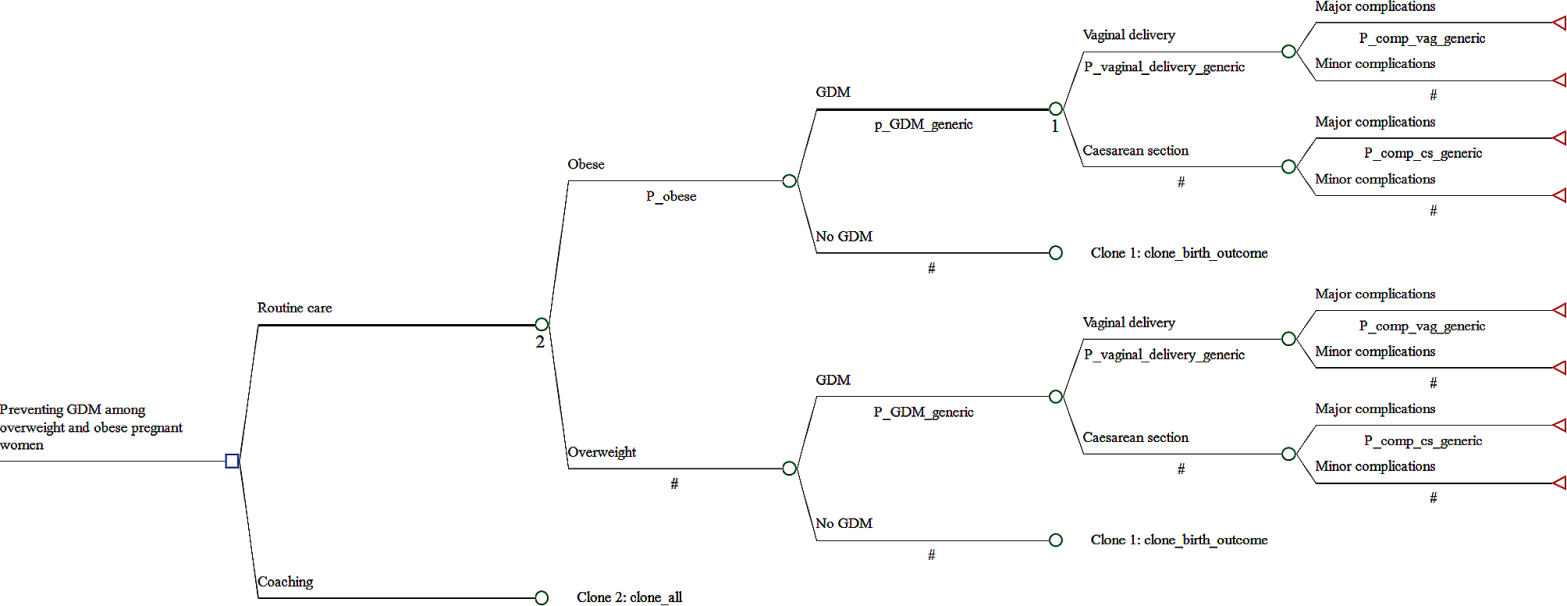
Simplified diagram of the decision tree model used to analyze the cost-effectiveness of coaching to prevent GDM

Figure 1 presents the model structure. The diagram shows that the routine care strategy considers two alternatives: the probability of having a BMI classified as being overweight or being obese during pregnancy. In the obese arm, pregnant women may develop GDM or not. Women were further stratified into type of birth using Australian Refined Diagnosis Related Groups (AR-DRG) codes. These are vaginal delivery minor complexity, vaginal delivery intermediate/major complexity, caesarean delivery minor complexity, and caesarean delivery intermediate/major complexity. The categorisation is influenced by the diagnoses meeting the ICD-10-AM criteria to code (10th Edition), the Diagnosis Complexity level (DCL) assigned to each code and the resultant Episode Clinical Complexity Score (ECCS). A minor delivery is associated with an ECCS of < 2, whereas an intermediate/major complexity is associated with a ECCS ≥ 2. In the no GDM arm of the obese chance node, the same chain of events has been considered outlined for the GDM arm (represented in clone 1). In the overweight arm of the routine care strategy, the flow of events was the same as described for the obese BMI arm (Fig. 1). In the alternative strategy, coaching, pregnant women follow exactly the same pathways depicted in the routine care strategy (represented in clone 2).

We examined the cost-effectiveness of the telehealth coaching intervention over routine care to prevent GDM by estimating the Incremental cost-effectiveness ratio (ICER). The ICER serves as a valuable tool in informing decision-making when evaluating the funding decision of a new drug or intervention. In Australia, there is no official statement regarding an explicit willingness-to-pay threshold for funding new medicines. However, the Pharmaceutical Benefits Advisory Committee (PBAC) tended to not recommend a drug for listing if the cost per additional year of life gained exceeded AUD 76,000. Conversely, they were unlikely to reject a drug if the cost per additional year of life gained was less than AUD 42,000 [32]. A newly developed medication that costs less than AUD 50,000 per Quality-Adjusted Life Year (QALY) gain is more likely to receive a recommendation for financial support in Australia [33].

We applied different thresholds of AUD 40,000 to 70,000 with an interval of AUD 10,000 assumed for decision-making to explore the influence on the incremental cost-effectiveness of coaching compared with routine care as part of sensitivity analyses.

### Costs

The data from 142 women referred to the program was available for costing the intervention. Costs included the staff time for the coaching intervention, costed at local wage rates (AUD 55.8/hour for a Senior Dietitian). The average cost of LWdP coaching intervention was AUD 311.

For costs of births, all babies birthed between 1 September 2016 to 31 August 2017 were included in the cost analysis. Costs associated with antenatal outpatient activity from 1 December 2015 and post-natal outpatient activity to 26 February 2018 were also included in the analysis [31].

Total costs for each type of birth per BMI category were calculated as the sum of mother and infant costs for respective hospital admissions and pre or post birth related outpatient appointments for the mother including appointments for gynaecology urology, continence, obstetrics complex pregnancy and midwifery lactation.

### Probabilities

The probability of having a major complexity versus a birth with minor complexity for each type of delivery (vaginal vs. caesarean) by GDM status were calculated for overweight and obese women using the proportion of births in each category from the cost analysis.

The LWdP program evaluation was not powered to detect a difference in the prevention of GDM. Therefore, the effectiveness of the coaching intervention at preventing GDM was determined from a systematic literature review of relevant similar interventions [16].

### Utilities

Utility scores were not collected in the LWdP program evaluation. Utility values for complex and uncomplex vaginal delivery and caesarean section were sourced from Kohler et al. (2018) [34] which reported quality of life weights for vaginal birth and caesarean section. Please refer to Supplementary Table 1 for the disutility associated with complex vaginal and caesarean delivery.

Ethical approval for the study was provided by the Royal Brisbane and Women’s Hospital Human Research Ethics Committee (HREC/17/QRBW/159) and was conducted according to the guidelines laid down in the Declaration of Helsinki.

## Results

**Fig. 2:**
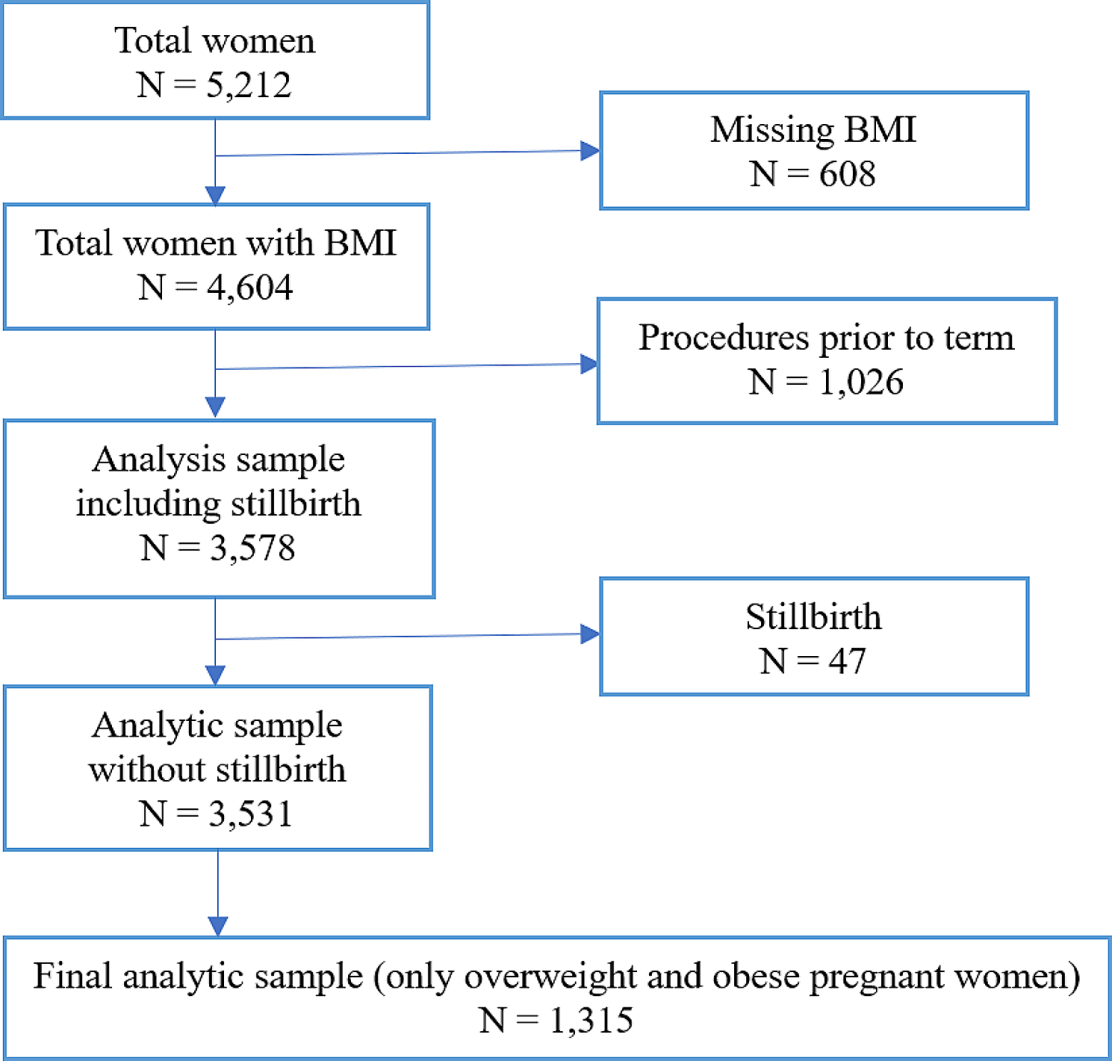
Analysis sample for costs and probabilities

Figure 2 briefly explains the flow of participants into the final analytic sample for costs and probabilities. Initially, the data set contained the information of 5,122 female participants. After exclusions, a total of 3,531 pregnant women were included in the subsample analyses. We restrict our sample to pregnant women who were either overweight or obese. The final analytic sample consisted of 1,315 pregnant women, with 51% classified as overweight and 49% classified as obese.

Table 1 reports the relative probabilities of the types of births including major and minor complexity by BMI category with 95% confidence intervals and associated costs. Women with GDM were more likely to experience major complexity during both vaginal and caesarean deliveries. There was also a higher proportion of having minor complexity during caesarean births for women with GDM. Costs were broadly similar between women classified as overweight and obese for type of delivery. For example, the costs of major complexity in vaginal delivery among overweight and obese pregnant women were AUD 30,498, and AUD 32,311, respectively.

Supplementary Table 1 presents the full list of variables used in the model including the cost of coaching, the cost of each category of birth outcomes, and the relative probabilities of different birth outcomes. Additionally, for sensitivity analysis purposes, Supplementary Table 1 presents the lower and upper values for each studied parameter for the one-way sensitivity analysis. A 30% change in the base values of each cost parameter has been considered to determine the lower and upper values, and 95% confidence intervals of the relative probabilities of various events are used. Further, supplementary Table 1 provides the distributions of the parameters used for performing probabilistic sensitivity analysis.

### Cost-effectiveness results

The base case cost-effectiveness result is presented in Table 2. The cost of the LWdP coaching intervention was lower than the routine care intervention, and the effectiveness of the LWdP strategy was slightly higher compared to the routine care strategy. As a result, the value of ICER turns to be negative, and the LWdP coaching intervention strategy is dominant.

**Fig. 3:**
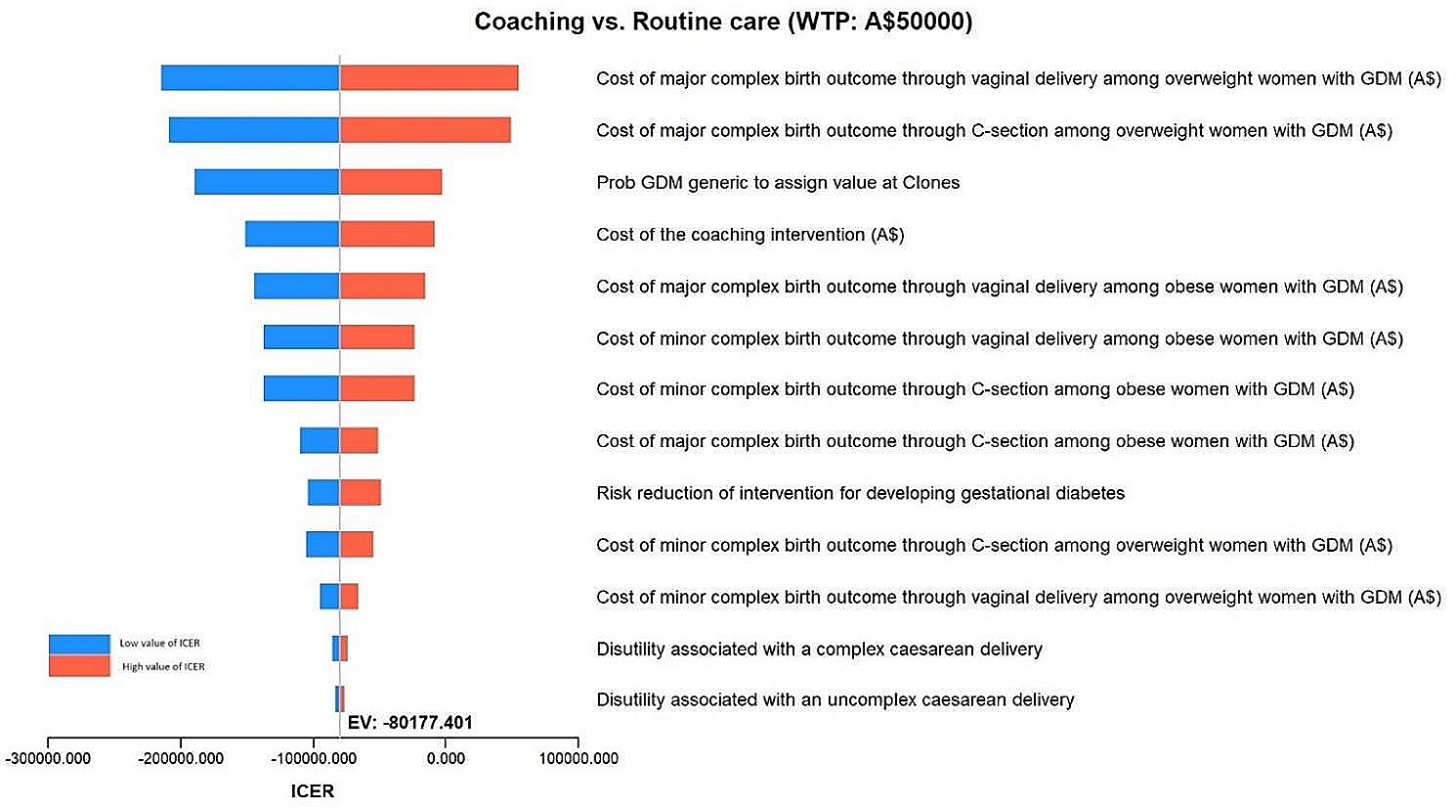
Tornado diagram - Incremental Cost Effectiveness Ratio

Multiple one-way sensitivity analyses of the cost-effectiveness of the LWdP coaching intervention against routine care in pregnant women is shown in Fig. 3. The tornado diagram shows a range of ICERs associated with the uncertainty range of most influential parameters. According to the tornado diagram, the value of ICER is sensitive to a number of parameters. The parameter that has the highest impact on ICER is shown on the top bar. For example, the sensitivity of ICER is highest to the costs of major complex birth outcome through vaginal delivery among overweight women with GDM, followed by costs of major complex birth outcome through C-section among overweight women with GDM. Additionally, the tornado diagram shows that the value of ICER is least sensitive to the value of disutility associated with an uncomplex caesarean delivery. The figure clearly shows that the value of ICERs increases if the value of all parameters increases.

**Table 1:** Costs and relative probabilities of birth outcomes through vaginal and caesarean delivery mode by BMI classification and GDM status

Delivery modes by minor and major complication birth outcomes	Obese		Overweight	
	GDM	No GDM	GDM	No GDM
**Probabilities**				
Any vaginal birth	0.54 (0.44, 0.65)	0.59 (0.53, 0.64)	0.56 (0.43,0.68)	0.61 (0.56, 0.67)
Major complex birth outcome vaginal delivery*	0.85 (0.78, 0.93)	0.64 (0.59, 0.70)	0.82 (0.72,0.91)	0.62 (0.57, 0.67)
Any caesarean section	0.46 (0.34, 0.57)	0.41 (0.35, 0.48)	0.44 (0.31,0.58)	0.39 (0.32, 0.45)
Major complex birth outcome caesarean section*	0.88 (0.81, 0.96)	0.67 (0.60, 0.73)	0.73 (0.61,0.85)	0.55 (0.48,0.61)
**Costs**				
Minor complexity vaginal birth	$11,577 ($4,653)	$10,302 ($4,417)	$11,324 ($3,571)	$11,170 ($5,221)
Major complexity vaginal birth	$19,170 ($25,602)	$19,961 ($32,001)	$23,305 ($35,274)	$17,434 ($27,376)
Minor complexity caesarean section	$15,712 ($4,113)	$15,936 ($4,370)	$16,360 ($3,408)	$15,137 ($4,154)
Major complexity caesarean section	$32,311 ($38,686)	$44,385 ($51,696)	$30,498 ($32,865)	$31,281 ($38,558)

Notes: (1) Abbreviation: GDM = Gestational Diabetes Mellitus. (2) All the cost values were rounded to the nearest whole number and were measured in Australian dollar. (3) Minor complexity birth probabilities are 1-this probability.

**Table 2:** Costs, effectiveness, and incremental cost-effectiveness estimates

Strategy	Cost (AUD)	Effectiveness	ICER
LWdP coaching intervention	20,828	0.894	Dominant
Routine care	20,933	0.893	
Incremental Cost / Effectiveness	-105	0.001	

Notes: 1. Abbreviation: ICER = Incremental cost-effectiveness ratio; AUD = Australian Dollar

**Fig. 4:**
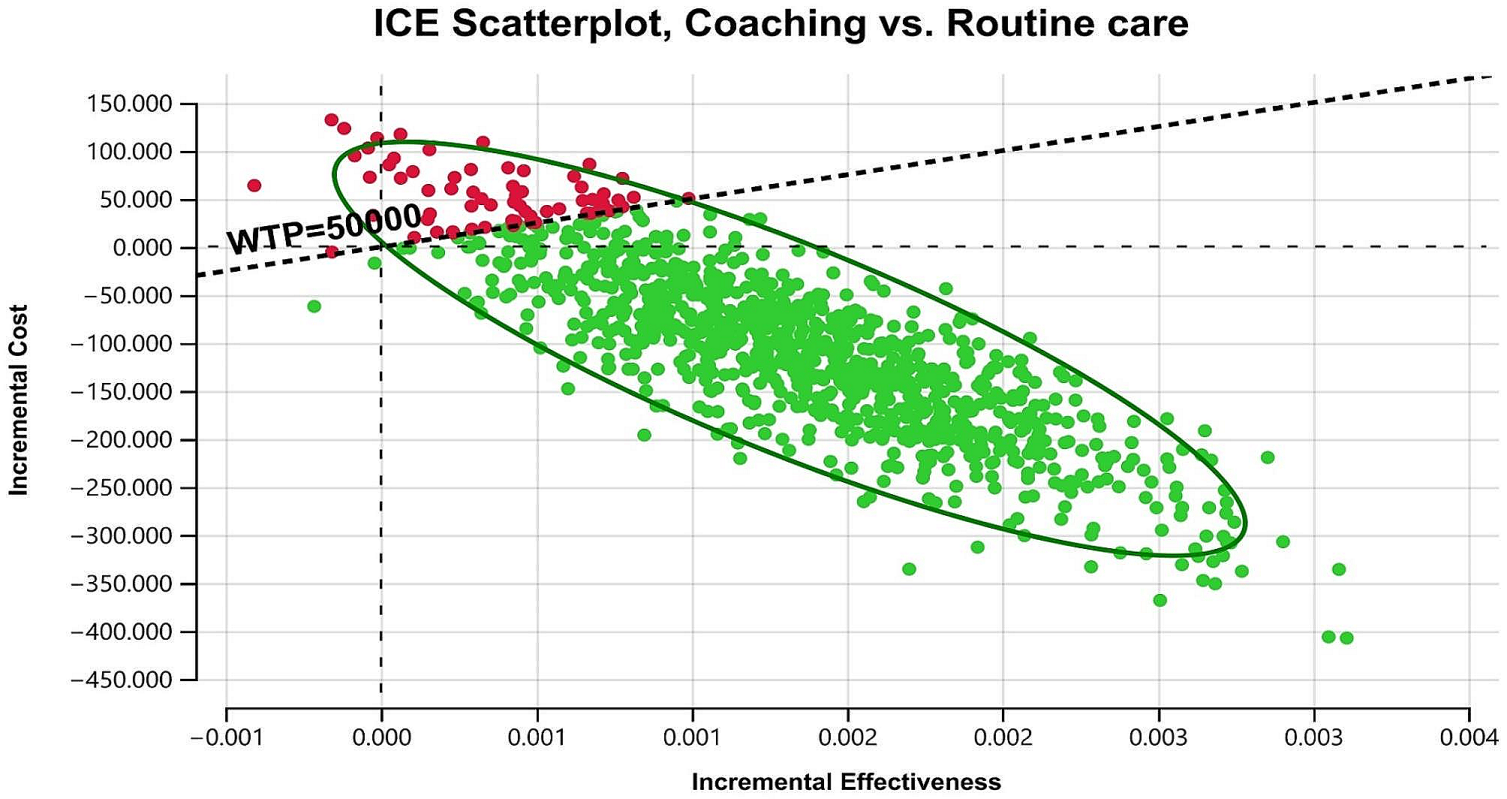
Monte Carlo simulations scatter plot of incremental cost-effectiveness of coaching compared with routine care with a willingness to pay (WTP) of AUD50, 000

Figure 4 displays the probabilistic sensitivity analysis using Monte Carlo simulation to estimate the cost-effectiveness of the coaching intervention versus routine care through 1,000 simulations. Each simulation generates a single outcome, unique ICER, represented as a green or maroon point on the plot. The ellipse represents the 95% confidence ellipse of outcomes. The ICE scatter plot showed that coaching was more effective and less costly in 93.60% of the trials using a willingness to pay (WTP) threshold of AUD 50,000. Additionally, we demonstrated how different thresholds may influence the incremental cost-effectiveness of coaching compared with routine care as part of sensitivity analyses. The ICE scatter plot (s) showed that coaching was more effective and less costly in over 90% of the trials when a threshold of AUD 40,000 to 70,000 with an interval of AUD 10,000 was assumed for decision-making (Supplementary Information Fig. 1). Therefore, coaching was an undominated cost-effective strategy.

## Discussion

This study aimed to evaluate the cost effectiveness of a dietitian delivered telephone lifestyle coaching program (LWdP) in preventing the risk of GDM in women with a BMI ≥ 25 kg/m^2^. A woman with GDM had a higher probability of a vaginal or caesarean delivery of intermediate/major complexity at each BMI category, increasing the cost of care. The analysis demonstrated that a coaching intervention that prevents GDM is likely to be cost saving to the health system when considering the immediate pregnancy and delivery costs.

Few studies have examined the cost effectiveness of lifestyle interventions to prevent GDM [35]. The findings of the current analysis are consistent with a recent Australian study examining lifestyle intervention for preventing GDM, hypertensive disorders of pregnancy or both [35]. Using a risk ratio for GDM from a large meta-analysis of intervention studies, Bailey and colleagues (2020) reported that lifestyle intervention to prevent GDM was cost neutral, and therefore likely cost effective, based on the cost associated with immediate pregnancy outcomes [35]. The findings are further supported by several studies examining the cost effectiveness of healthy eating (with or without physical activity) interventions to prevent excess gestational weight gain [36, 37]. While behavioural interventions for pregnant women are associated with higher costs to deliver the additional care [37], structured behavioural interventions emphasising healthy eating with physical activity are preferable and cost-effective for reducing gestational weight gain [20, 36, 38].

In contrast to these findings, a behavioural intervention based on diet and physical activity targeting pregnant women with a BMI of ≥ 30 was deemed not cost-effective when compared to standard care in improving QALYs in UK [39]. However, the authors of this study did not include pregnancy delivery costs, with follow up until 36 weeks gestation included [39].

The findings of the present study need to be considered in the context of several strengths and limitations. A comprehensive decision analytic model (decision tree) was developed to capture the therapeutic and financial implications of the cost-effectiveness of the LWdP coaching intervention in preventing GDM. The study considered utility value as the outcome measure in the model with most of the parameters (e.g., costs of the intervention and probabilities) estimated using real world data from Australian pregnant women to boost validity and reliability of the results.

However, this analysis was carried from the Australian healthcare system perspective and might not be generalizable to other countries with different healthcare systems. Furthermore, the value of the intervention effectiveness, utility, and disutility weights associated with caesarean delivery were extrapolated from the existing literature and may not reflect the cultural, and environmental differences in the Australian context. However, there are some constraints that cannot be avoided when modelling reality with a decision-analytic model. While the cost savings are modest, there could be substantial ongoing benefits to the mother and child from avoidance of birthing complications and improvements in health behaviours that are not able to be captured in the decision tree model as this model only captures costs and effects around the pre-birth, time of birth and limited follow up (6 months post- delivery time horizon) at the birthing institution. It is well established that dietary intake and gestational weight gain during pregnancy impact not only on pregnancy outcomes [1, 40], but on the long term health of mothers and their offspring [41]. Intervening during pregnancy when women are in contact with the health system, and motivated for change is likely to have long term benefits in terms of preventing obesity, diabetes, and cardiovascular disease for both woman and offspring [42]. These future cost savings need to be considered in economic modelling. Future research should explore alternative and hybrid delivery models for intervention during pregnancy, and the long-term health benefits of nutrition intervention from the perspective of the woman, offspring and family should be included in future cost-effectiveness studies.

Women with GDM during pregnancy were much more likely to have births– whether vaginal delivery or caesarean of increased complexity. The cost of delivery with increased complexity was much higher than without. Therefore, preventing GDM is likely to reduce complications during birth, subsequently giving both mother and baby a better experience and start to life as well as reducing costs to the health system.

## Conclusion

This study adds to the growing body of evidence that evidence-based lifestyle interventions that focus on supporting nutrition behaviour change are cost-effective in preventing GDM from the Australian healthcare system perspective. Taking a life course approach to preventive health care, focussing on supporting women developing healthy eating behaviours prior to conception and in early pregnancy is likely to have a long-term positive health and economic impact on women and that of her offspring. Healthcare policy and decision makers need to consider telehealth and broad-reach delivery of structured lifestyle interventions during pregnancy to lower short term costs associated with GDM to the health system. Additionally, long-term cost savings are likely but require further investigation.

### Electronic supplementary material

Below is the link to the electronic supplementary material.

**Supplementary Table 1**: Summary of input parameters for the cost-effectiveness model

## Data Availability

The datasets analysed for the current study are available from the corresponding author on reasonable request and in accordance to local restrictions governing the privacy of information obtained from medical records.
