# Supplementary material for: A cost-effectiveness evaluation of a dietitian-delivered telephone coaching program during pregnancy for preventing gestational diabetes mellitus
Source: Cost Eff Resour Alloc. 2024 Mar 1;22:18. doi: 10.1186/s12962-024-00520-9 (PMC10908067; doi:10.1186/s12962-024-00520-9)
Supplement: Supplementary file 1 — Supplementary Table 1: Summary of input parameters for the cost-effectiveness model [file 12962_2024_520_MOESM1_ESM.docx]

Supplementary Table 1: Summary of input parameters for the cost-effectiveness model

| **Parameters** | **Base case** | **Sensitivity Analysis** | | **Distribution type*** | **Data Source** |
| --- | --- | --- | --- | --- | --- |
|  |  | **Low** | **High** |  |  |
| Cost of the coaching intervention, Mean | 311 | 218 | 404 | Gamma | Primary |
| Cost of major complex birth outcome through C-section among obese women with GDM, Mean (SD) | 32,311 (38,686) | 22,618 | 42,004 | Gamma | Primary |
| Cost of minor complex birth outcome through C-section among obese women with GDM, Mean (SD) | 15,712 (4,113) | 10,998 | 20,426 | Gamma | Primary |
| Cost of major complex birth outcome through vaginal delivery among obese women with GDM, Mean (SD) | 19,170 (25,602) | 13,419 | 24,921 | Gamma | Primary |
| Cost of minor complex birth outcome through vaginal delivery among obese women with GDM, Mean (SD) | 11,577 (4,653) | 8,104 | 15,050 | Gamma | Primary |
| Cost of major complex birth outcome through C-section among overweight women with GDM, Mean (SD) | 30,498 (32,865) | 21,349 | 39,647 | Gamma | Primary |
| Cost of minor complex birth outcome through C-section among overweight women with GDM, Mean (SD) | 16,360 (3,408) | 11,452 | 21,268 | Gamma | Primary |
| Cost of major complex birth outcome through vaginal delivery among overweight women with GDM, Mean (SD) | 23,305 (35,274) | 16,313 | 30,297 | Gamma | Primary |
| Cost of minor complex birth outcome through vaginal delivery among overweight women with GDM, Mean (SD) | 11,324 (3,571) | 7,927 | 14,721 | Gamma | Primary |
| Disutility associated with a complex caesarean delivery | -0.13 | -0.137 | -0.124 | Normal | (Kohler et al., 2018) |
| Disutility associated with an uncomplex caesarean delivery | -0.11 | -0.116 | -0.104 | Normal | (Kohler et al., 2018) |
| Disutility associated with a complex vaginal delivery | -0.04 | -0.006 | -0.002 | Normal | (Kohler et al., 2018) |
| Probability of major complex birth outcome through C-section among obese women with GDM | 0.88 | 0.81 | 0.96 | Beta | Primary |
| Probability of major complex birth outcome through vaginal delivery among obese women with GDM, Mean (SD) | 0.85 | 0.78 | 0.93 | Beta | Primary |
| Probability of being obese | 0.49 | 0.47 | 0.52 | Beta | Primary |
| Probability of having GDM among obese women | 0.23 | 0.17 | 0.30 | Beta | Primary |
| Probability of having GDM among overweight women | 0.18 | 0.11 | 0.24 | Beta | Primary |
| Probability of vaginal delivery among obese women with GDM | 0.54 | 0.44 | 0.65 | Beta | Primary |
| Probability of vaginal delivery | 0.67 | 0.58 | 0.75 | Beta | Primary |
| Risk reduction of intervention for developing gestational diabetes | 0.39 | 0.32 | 0.46 | Beta | (Teede et al., 2022) |
| Utility value for uncomplex vaginal delivery | 0.96 | 0.95 | 0.98 | Beta | (Kohler et al., 2018) |

Notes: 1. Abbreviation: C-section = Caesarean section; SD = Standard Deviation.  2. * Indicates that distribution was used for the probabilistic sensitivity analysis. 3. All the cost values were rounded to the nearest whole number. 4. All the probability values were rounded to 2 decimal places.


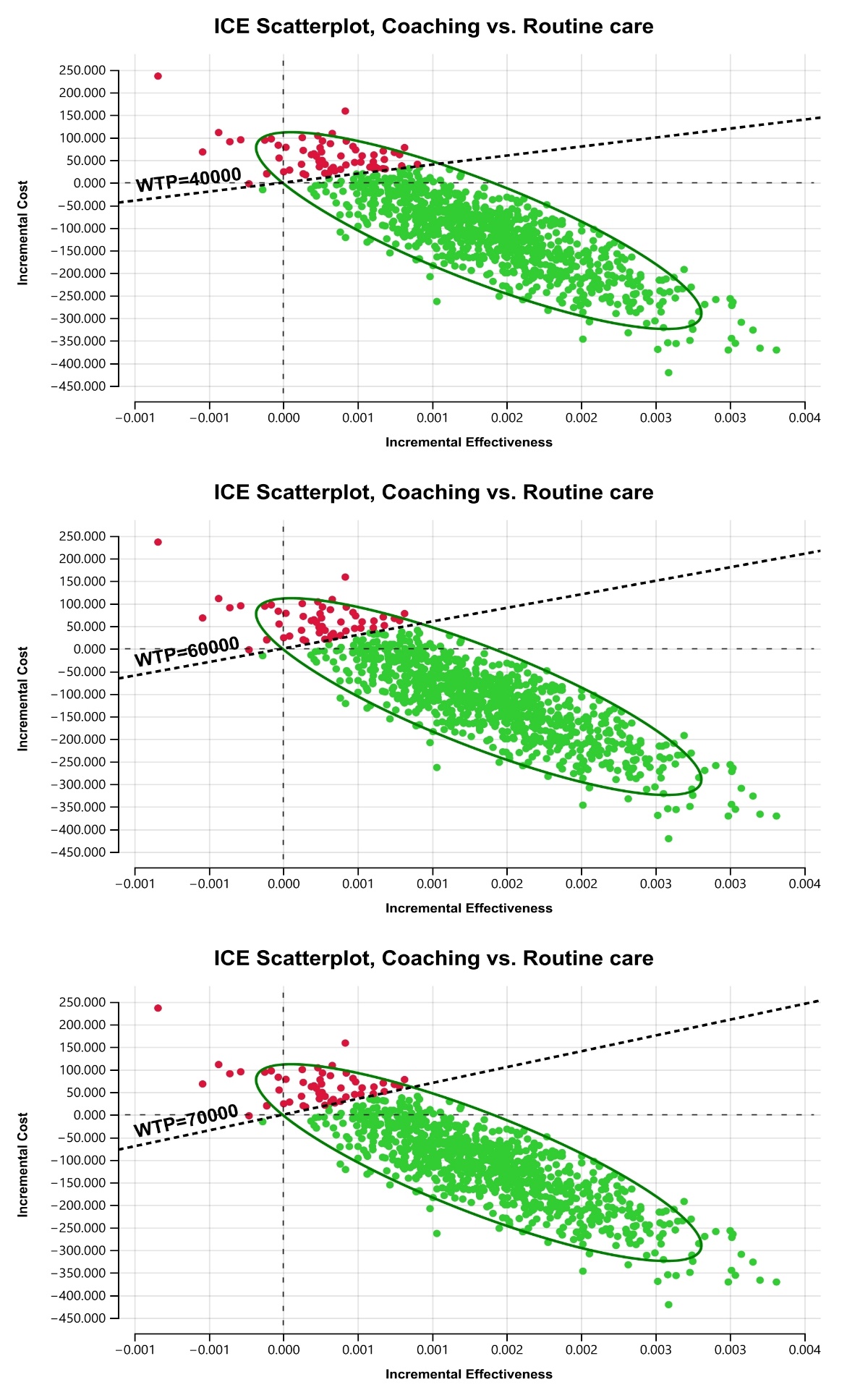


Supplementary Figure 1: Monte Carlo simulations scatter plot of incremental cost-effectiveness of coaching compared with routine care with a willingness to pay (WTP) of AU$40 000, AU$60 000 and AU$70 000.
